# Temperature sensitivity and enzymatic mechanisms of soil organic matter decomposition along an altitudinal gradient on Mount Kilimanjaro

**DOI:** 10.1038/srep22240

**Published:** 2016-02-29

**Authors:** Еvgenia Blagodatskaya, Sergey Blagodatsky, Nikita Khomyakov, Olga Myachina, Yakov Kuzyakov

**Affiliations:** 1 Dept. of Soil Science of Temperate Ecosystems, University of Göttingen, 37077 Göttingen, Germany; 2 Institute of Physicochemical and Biological Problems in Soil Science, Russian Academy of Sciences, 142290 Pushchino, Russia; 3 Institute of Plant Production and Agroecology in the Tropics and Subtropics, University of Hohenheim, 70593 Stuttgart, Germany; 4 Forensic Science Centre of the Ministry of Internal Affairs of the Russian Federation, 125130 Moscow Russia; 5 Institute of General and Inorganic Chemistry, 100170 Tashkent, Uzbekistan; 6 Department of Agricultural Soil Science, University of Göttingen, 37077 Göttingen, Germany

## Abstract

Short-term acceleration of soil organic matter decomposition by increasing temperature conflicts with the thermal adaptation observed in long-term studies. Here we used the altitudinal gradient on Mt. Kilimanjaro to demonstrate the mechanisms of thermal adaptation of extra- and intracellular enzymes that hydrolyze cellulose, chitin and phytate and oxidize monomers (^14^C-glucose) in warm- and cold-climate soils. We revealed that no response of decomposition rate to temperature occurs because of a cancelling effect consisting in an increase in half-saturation constants (K_m_), which counteracts the increase in maximal reaction rates (V_max_ with temperature). We used the parameters of enzyme kinetics to predict thresholds of substrate concentration (S_crit_) below which decomposition rates will be insensitive to global warming. Increasing values of S_crit,_ and hence stronger canceling effects with increasing altitude on Mt. Kilimanjaro, explained the thermal adaptation of polymer decomposition. The reduction of the temperature sensitivity of V_max_ along the altitudinal gradient contributed to thermal adaptation of both polymer and monomer degradation. Extrapolating the altitudinal gradient to the large-scale latitudinal gradient, these results show that the soils of cold climates with stronger and more frequent temperature variation are less sensitive to global warming than soils adapted to high temperatures.

While soil organic carbon (C) decomposition is generally thought to increase with temperature^1^,^2^, recent studies have postulated that this may be a temporary effect, potentially mitigating the expected soil C losses due to climate change^3^,^4^,^5^. “Thermal adaptation” has been defined as an adaptation of microbial heterotrophic activity, e.g. by a decrease in heterotrophic soil respiration rate per unit microbial biomass in response to a sustained temperature increase^6^. This definition provides a convenient conceptual basis for characterizing thermal adaptation phenomena and developing further hypotheses, although soil organic matter (SOM) decomposition is a more complex process than microbial respiration *per se*.

The multistage decomposition of SOM and ultimate CO_2_ release depend on the combined response of extra- and intracellular, enzymatically mediated reactions to temperature^7^. The extracellular steps of SOM decomposition include 1) the release of monomers from polymeric compounds, and 2) the active and passive transport of monomers into microbial cells. The subsequent intracellular enzymatic reaction chain includes the catabolism of consumed organic substances, releasing CO_2_ as the end-product. Both the extra- and intracellular enzymatic steps may play key roles in the SOM response to global warming, but with different sensitivities to temperature change^8^. This calls for considering them separately when analyzing the temperature response of SOM decomposition, and indicates that a more mechanistic understanding of thermal adaptation is needed to predict soil carbon responses to climate change. Assuming that the degradation of recalcitrant compounds arises from higher activation energies for these reactions than for more labile substances, the Arrhenius equation would predict higher temperature sensitivity for decomposition rates (as a relative increase when warming) as the molecular complexity of the substrate increases^9^,^10^. Consequently susceptibility to global warming should be higher for soils with larger proportions of recalcitrant OM^11^.

Three mechanisms have been proposed to explain thermal adaptation: 1) change in the substrate affinity of enzyme systems (i.e. in K_m_)^12^, which may reflect the shifts in microbial community structure^13^,^14^, 2) the reduction of soil microbial biomass and enzyme expression at higher temperatures^4^,^15^, which can be linked with changes in microbial physiology, and 3) changes in quantity and quality of substrate, affecting reaction rates of enzyme-catalyzed processes^16^,^17^. The first explanation assumes that changes in microbial community structure, such as the bacterial-to-fungal ratio^18^, and corresponding shifts in enzyme catalytic pathways can cause dramatic changes to the rates of enzyme-mediated reactions under a warmer climate^19^,^20^. Climate-induced shifts in microbial community structure can affect the characteristics of enzyme systems. For example, enzymes of an altered community may be capable of more rapid conformational changes. Potential acceleration of the reaction rate due to an enzyme’s higher structural flexibility can be counterbalanced, however, by a reduced affinity of the more flexible enzyme to substrate (increased K_m_) with increasing temperature. It still remains to be tested whether more flexible or more stable enzymes systems with lower temperature sensitivity of K_m_ will be benefitting as a consequence of global warming^12^.

The second explanation is based on microbial physiology and assumes a decrease in microbial carbon use efficiency (CUE) with warming^4^,^21^, reducing enzyme production and/or decreasing enzymatic activity. However, hypothesized decreases in CUE under warming are controversial with little^22^ to no^23^,^24^ experimental evidence and a weak theoretical basis^12^. Furthermore, microbial CUE can show a variable response to warming, due to interactive effects of temperature and substrate quality (e.g. C:N ratio), which control the activity of specific microbial groups and the production of extracellular enzymes^25^.

The third thermal adaptation mechanism (reduction of available substrate) refers to the relationship between the substrate amount and the rate of CO_2_ production in SOM decomposition. Warming can affect both the amount of decomposing substrate and its’ quality (susceptibility to decomposition)^28^. Accordingly, microbial responses to temperature increase may diverge strongly from the predictions based solely on the chemical properties (activation energy) of SOM. Therefore, an explanation complementary to the Ahrrenius and substrate quality concepts^10^ was suggested on the basis of Michaelis–Menten kinetics^29^:


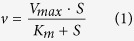


Remarkably, in addition to the influence of substrate concentrations, this equation also relates the rates of enzyme-catalyzed reactions with functional parameters of the microbial community: maximal reaction rate (V_max_) and enzyme-to-substrate affinity (K_m_). Accordingly, temperature response of reaction rate *v* is a result of simultaneous changes of independent variable (substrate S) and two parameters (V_max_ and K_m_) in the equation 1. Prolonged warming usually causes substrate exhaustion and decrease in reaction rate *v*. The increase of V_max_ with temperature accelerates the decomposition rate, especially at high substrate concentrations. In contrast, the increase of K_m_ with temperature would slow the reaction rate, and this effect is especially pronounced at low substrate concentrations. This “canceling effect” (due to larger K_m_ in the denominator of Eq. 1) reduces the sensitivity of the reaction rate to temperature. Consequently, final CO_2_ production may be unaffected by temperature^30^. To our knowledge, this theoretically predicted canceling effect has never been proven simultaneously for exo- and endocellular enzymatic reactions in the same experiment. Disentangling the interactive effects of thermal adaptation mechanisms on the *in situ* enzyme reaction rates requires determining whether V_max_ or K_m_ is responsible for altered process rates at increasing temperatures^19^,^30^.

We investigated the temperature dependency of the Michaelis-Menten parameters V_max_ and K_m_ by determining these parameters at 10 and 20 °C. This was done for the decomposition of substrates with contrasting chemical lability. De-polymerization of macromolecular substrates was studied based on the activity of three extracellular hydrolytic enzymes: β-1,4-glucosidase (responsible for cellulose decomposition, releasing glucose units from the ends of cellulose chains), N-acetylglucosaminidase (degrades chitin and peptidoglycan, polymers of fungal and bacterial origin, respectively) and acid phosphatase (releases available P from organic compounds, e.g., phytate).

The decomposition of easily available substrates within microbial cells was simulated by mineralization of ^14^C-labeled glucose. Glucose is a monomer of cellulose and hemicellulose – the main source of organic input in soil. ^14^C labeling allowed the temperature response to be assessed at very low substrate levels (starting from 35 nmol glucose g^−1^ soil). An advantage of our methodology was the determination of immediate mineralization of ^14^C-labeled glucose to ^14^CO_2_ (within 15 minutes after glucose application). Neither the small amounts of glucose added nor the short time span were sufficient for microbial growth. Thus, added glucose was catabolized by the microorganisms currently active in soil.

There is no consensus regarding thermal adaptation of microbial SOM decomposition under different climatic conditions. It is still controversial whether soils adapted to warmer temperatures are less responsive to warming compared to soils from colder environments^11^,^31^. We hypothesized weaker responses to temperature increases from microorganisms adapted to stronger temperature variations. To study the responses of soils developed under different climatic conditions^12^ and to compare thermal adaptation capabilities in microbial communities from different temperature regimes, we sampled soils along the altitudinal gradient on Mt. Kilimanjaro (950, 2010, 2435, 2780 and 3020 m a.s.l.). The gradient on this mountain is well suited for thermal adaptation studies because the soils (all Andosols) developed from identical parent material (volcanic ash) during the same time (<360 ky). A key factor is that not only are the mean annual temperatures lower, but (depending on the season) the diurnal temperature amplitudes are up to 1.5–2 times larger for soils from high versus low altitudes^32^. Therefore, the thermal adaptation capabilities of the microbial communities and associated enzyme systems, developed over the long term under contrasting climatic conditions, can be compared^21^. This altitudinal gradient, however, does not directly correspond to common climate warming scenarios, which assume that overall warming is accompanied by stronger temperature fluctuations. Since vegetation changes with altitude, affecting soil C content and microbial activity^33^, we did not compare absolute values of enzymatic and respiratory activities. Instead, only relative temperature responses were considered, expressed as the Q_10_ values (i.e. ratio of corresponding parameters at 20 and 10 °C).

## Results

### Temperature sensitivity of extracellular de-polymerization vs glucose mineralization

**Figure 1:**
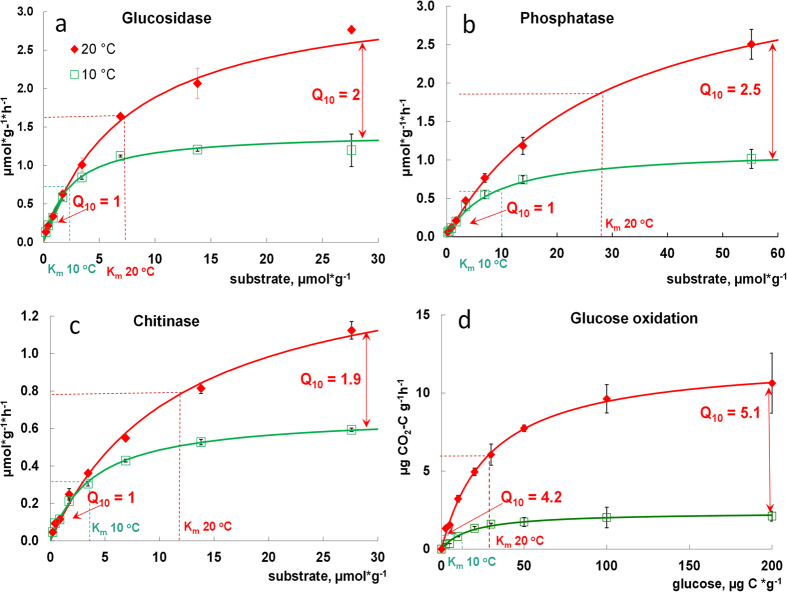
Rates of reactions mediated by hydrolytic enzymes (**a–c**) and rates of glucose oxidation to CO_2_ (**d**) as dependent on substrate concentration at 10 and 20 °C for the site located at 2010 m a.s.l. Symbols – experimental data, lines – approximation by Michaelis–Menten kinetics (Eq. 1). Bars show standard deviations of the means (n = 3).

**Figure 2:**
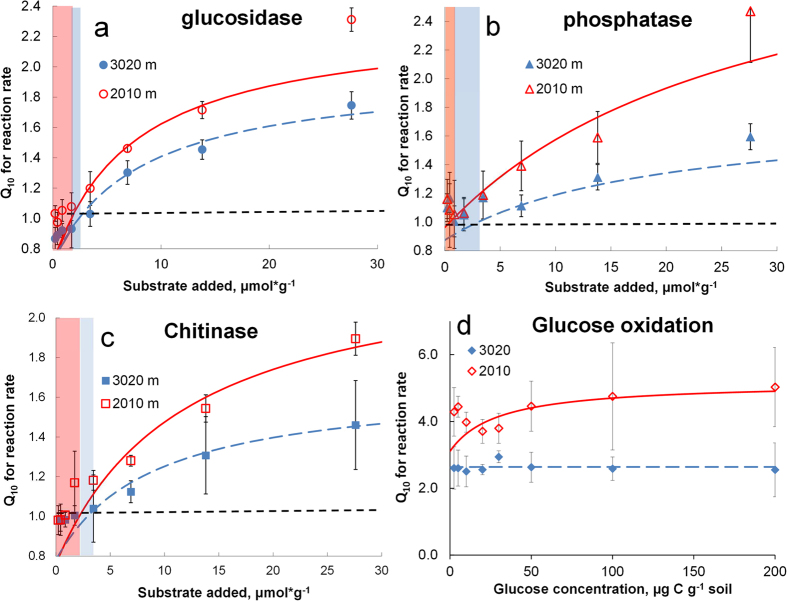
The Q_10_ values for enzymatic activities (**a–c**) and glucose oxidation to CO_2_ (**d**) as dependent on substrate concentration at two altitudes. The emphasized sections show the concentration range at which no temperature effects occur (below S_crit_) with shading colors corresponding to different altitudes. The Q_10_ values derived from experimental data are shown as symbols. The model simulations based on experimentally obtained parameters of Michaelis–Menten kinetics (Eqs 1 and 1S) are shown as curves (**a–c**). For glucose oxidation (**d**) at 3020 m elevation, non-linear trend was very weakly expressed. Bars show standard deviations of the means (n = 3).

To elucidate the canceling mechanisms, we compared the K_m_ and V_max_ for three extracellular enzymes and for intracellular glucose oxidation at two temperatures (10 and 20 °C). The higher temperature caused a 25–42% larger increase of K_m_ than of V_max_ for the three exo-enzymes, i.e., Q_10_^Km^ > Q_10_^Vmax^. This led to canceling at a low substrate level (Table 1, Fig. 1a–c). For example, for soil from 2010 m a.s.l. the higher temperature caused a 2.3-fold increase in the V_max_ for β-1,4-glucosidase, but an even stronger 3.3-fold increase of K_m_. This prevented an increased enzymatic reaction rate for low substrate concentrations (Fig. 1a). Such an increase in K_m_, however, was insufficient for a canceling effect at high substrate levels. Therefore, the Q_10_ for overall reaction rate (Q_10_^total^) of depolymerization reactions increased with increasing substrate amounts (Fig. 2a–c, Eq. 1S).

We used Equation 2 (for derivation, see Supplement 2) to determine the substrate concentration threshold (S_crit_) below which the canceling effect occurs and no positive response of reaction rate to temperature is detected:


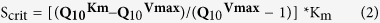


This threshold increases with increasing difference between the temperature sensitivities of K_m_ and V_max,_ [**Q**_**10**_^**Km**^ − **Q**_**10**_^**Vmax**^], but the influence of this difference is lower at high values of **Q**_**10**_^**Vmax**^. The S_crit_ is therefore an indicator of thermal adaptation by enzyme systems, i.e. a larger S_crit_ means stronger thermal adaptation, reflected in the wider range of substrate concentrations over which the system is unresponsive to temperature increases. At substrate concentrations below 1.68 μmol g^−1^ soil (similar to natural concentrations of cellulosic compounds^34^,^35^) no significant temperature response of enzyme activities were observed for the cellulose degrading enzymes (Supplementary Table S3, Fig. 2a). At higher substrate levels (>6 μmol g^−1^), the higher temperature stimulated increased activity of all tested enzymes.

In contrast to extracellular depolymerization, the rate of intracellular glucose mineralization differed substantially between 10 and 20 °C for all tested concentrations (Fig. 1d, Supplementary Fig. S5) and the canceling effect was not detected, even at low substrate levels. The temperature response of monomer oxidation showed a strongly accelerated reaction rate instead of a canceling effect (Fig. 2d, Supplementary Fig. S1) with Q_10_^total^ of 2.5 to 5 and Q_10_^Km^ < Q_10_^Vmax^. This occurred at a substrate range similar to that for which polymeric compound degradation displayed canceling (0.03 to 2.8 μmol glucose g^−1^). At saturating substrate concentrations, both extracellular depolymerization and monomer oxidation to CO_2_ were highly responsive to temperature. Nonetheless, the 2.5- to 5-fold increase of glucose mineralization rates caused by warming was always greater than the increases in depolymerization rates, which never exceeded a factor of 2.5 (Fig. 2).

### Thermal adaptation of enzymatic activity and glucose mineralization

**Figure 3:**
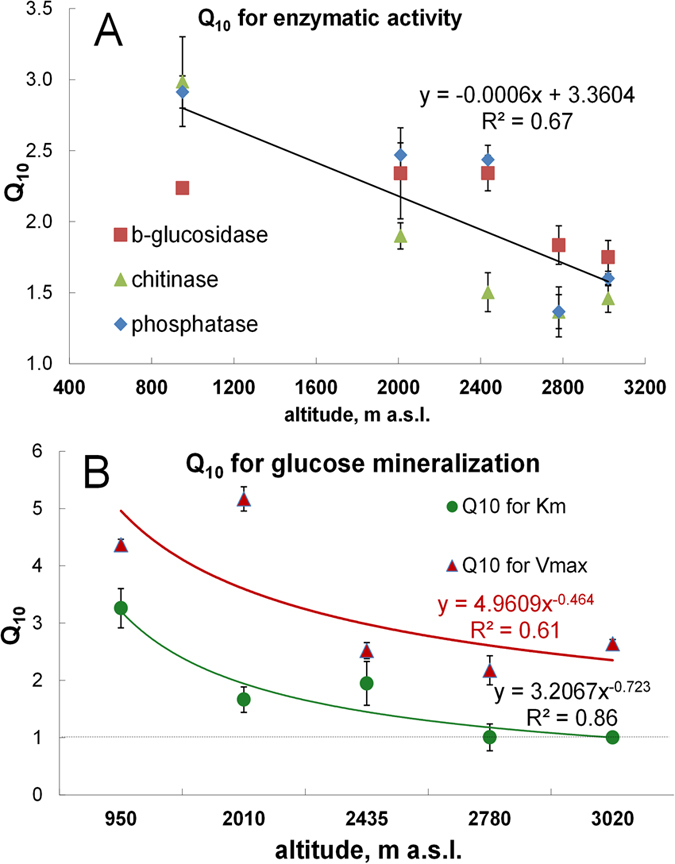
The **Q**_**10**_^**total**^ values for hydrolytic enzyme activity at saturating substrate concentrations (**A**) and the increase in V_max_ and K_m_ induced by a temperature increase from 10 to 20 °C for ^14^C-glucose oxidation (**B**) depending on altitude. Symbols – experimentally derived values for **Q**_**10**_^**total**^ (**B**), **Q**_**10**_^**Vmax**^, and **Q**_**10**_^**Km**^ (**A**). Lines are the trend-lines obtained by the best fitting of power (**A**) and linear functions (**B**) at P values < 0.05, bars show standard deviations of the means (n = 3).

**Figure 4:**
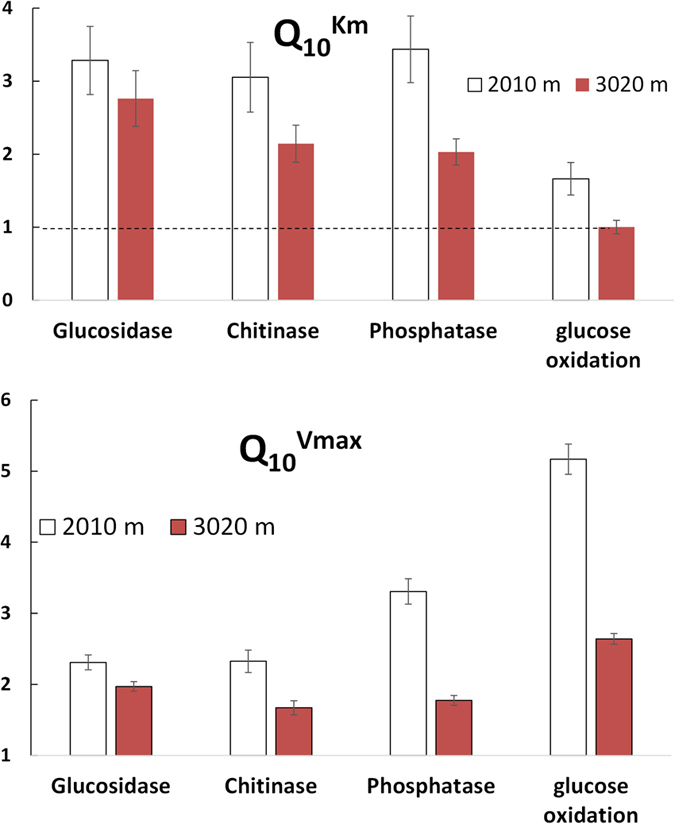
The values of **Q**_**10**_^**Km**^ (**a**) and **Q**_**10**_^**Vmax**^ (**b**) for hydrolytic reactions and for reactions of glucose oxidation at low and high altitudes. Bars show standard deviations of the means (n = 3).

In order to estimate the thermal adaptation capability of enzyme systems to prevailing temperature regimes, we compared the responses of exoenzyme activities to temperature (Q_10_^total^) in warm- (2010 m a.s.l.) versus cold-adapted (3020 m a.s.l.) soils (see Methods section). We also examined the Q_10_ values of K_m_ and V_max_ for intra- and extracellular enzymes at 2010 and 3020 m a.s.l. The Q_10_^total^ of reaction rates at concentrations higher than S_crit_ were always lower at high altitude (3020 m) compared to soils from lower sites (2010 m) (Figs 2 and 3A). The S_crit_ values of depolymerization reactions were 35–42% larger at higher altitudes, despite the lower C_org_ content of these soils (Table S3; Fig. 2a–c, emphasized sections). Both Q_10_^Km^ and Q_10_^Vmax^ were lower at high altitude (Fig. 4a,b). Thus, hydrolytic enzymes responded less intensively to temperature differences at higher altitudes. This indicates a larger compensatory response of microbial enzyme systems at higher altitudes, associated with the larger and more frequent temperature variations there^32^.

The thermal adaptation capability of enzymes mineralizing glucose was tested at 5 sites along the altitudinal gradient. At the three lowest altitudes (950, 2010, 2435 m a.s.l.) intracellular enzyme systems responded to the higher temperature with strongly increased K_m_ values (Q_10_^Km^ = 3.3, 1.7, 1.9, respectively, Supplementary Table S4) indicating an occurrence of more structurally flexible enzyme systems^12^. Remarkably, at high altitudes (2780 and 3020 m) no change in substrate affinity for a 10 °C temperature difference was observed for glucose oxidation, demonstrating temperature stability of the intracellular enzyme systems (Q_10_^Km^ = 1, Fig. 3B, Supplementary Table S4). However, the V_max_ of glucose mineralization was 2 times less sensitive to temperature at altitudes of 2435, 2780 and 3020 m as compared to 950 and 2010 m (Fig. 3B). Thus, microorganisms at higher altitudes strongly facilitated glucose decomposition in response to temperatures increase, when substrate concentrations were low. However, this was accompanied by a relative retardation of enzyme activity at substrate excess (smaller increase in V_max_). Consequently, the Q_10_^total^ for glucose oxidation was lower at 3020 m versus 2010 m altitude and was independent of substrate amount (Fig. 2d).

## Discussion

The higher temperature strongly accelerated mineralization of glucose in soil, but did not alter the decomposition rate of polymers at concentrations below the relevant S_crit_. Below this value, the Q_10_^Km^ > Q_10_^Vmax^ values resulted in a canceling effect. Thus, at a substrate concentration below 1.68 μmol g^−1^ soil, depolymerization was the process determining the temperature sensitivity of substrate decomposition. Decomposition of polymers to monomers was always found to be less temperature sensitive than the mineralization of low molecular weight soluble substrate (glucose), seemingly contrary to predictions based on the Arrhenius equation. Below we present theoretical explanations of this behavior considering 1) the particle diffusion that lead to biochemical reactions; 2) changes in apparent activation energy of enzyme-catalysed reactions in soil; and 3) temperature sensitivity of enzyme-substrate complexes.

Temperature acceleration of Brownian motion increases the probability of collision between substrate and enzyme. The relative increase in diffusion rates with temperature is greater for smaller substrates such as glucose than for high molecular weight compounds, i.e. SOM colloids and enzymes^36^.

Lower temperature sensitivity of decomposition of polymeric compounds versus their more labile monomers, as observed in our study, is explained by enzymatic mechanisms rather than by the Arrhenius equation and differences in activation energy. According to the Arrhenius function^10^, the temperature sensitivity of less labile organics should be higher than that of more labile compounds due to the higher activation energy of the former^9^,^10^. Indeed, the activation energy (E_a_) for glucose oxidation (about 50 kJ mol^−1^) ^10^ is much lower than that for glucose release from cellobiose (oligomer of cellulose, 80–130 kJ mol^−1^)^37^ or from cellulose (109–210 kJ mol^−1^)^38^. Note, however, that the activation energy determined during chemical decomposition of cellulose by pyrolysis or by hydrolysis with sulfuric acid does not correspond to soil conditions, where the reaction is enzyme-catalysed. In the presence of cellulases (e.g. β-glucosidase) the activation energy for cellulose (e.g. 3–50 kJ mol^−1^)^37^ could be comparable or even much lower than the E_a_ for glucose oxidation. This theoretical prediction corresponds well to similar or lower values of apparent activation energy obtained in our study for reactions of glucose oxidation as compared with the decomposition of polymers (Tables 1, S4). Thus, the apparent temperature response does not fully represent predictions based on inherent activation energy. Temperature sensitivity of polymer decomposition is mainly dependent on other factors such as substrate availability^17^,^39^,^40^, enzyme production^4^ or affinity of enzymes to substrate^10^. The comparison of temperature sensitivity of ^14^C-glucose mineralization with cellulolytic activity in homogenized soil suspension requires certain caution as enzyme activity in suspension is much higher than those in intact soil. According to our results, however, the difference in temperature sensitivity between hydrolytic enzymes and respiration seems to be even higher when both are compared in undisturbed soil. Further research could be directed to comparison of utilization of N- and P-containing monomers with activity of corresponding enzymes, thus linking temperature regulation with possible stoichiometric constraints.

A more detailed analysis of the reaction processes yields further understanding in terms of the thermal stabilities of enzyme-substrate complexes. Simple enzymatic reactions lead to the *reversible* formation of enzyme-substrate complexes (ESC). These complexes can be further decomposed into free enzymes (E) and products (P) or dissociate back to enzymes and substrates (S)^41^. Each step of the enzyme-catalyzed reaction is governed by the respective rate constants which are related to kinetic parameters K_m_ and V_max_:


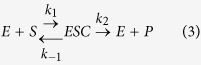


For ESC formation at steady-state:


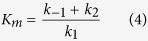



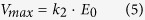


Thus, a K_m_ increase with temperature implies that the rate of ESC formation (k_1_) is less temperature sensitive than ESC dissociation (k_−1_ + k_2_). Assuming that at steady-state k_1_ ≈ k_−1_, this also indicates a much smaller increase in k_1_ than in k_2_, which is the rate constant for product formation. Therefore, larger Q_10_^Km^ values indicate that ESC dissociation responds to temperature more strongly than does ESC formation, thus demonstrating the mechanism of temperature flexibility of enzyme systems^12^. Assuming the same enzyme expression at both temperatures, consistently larger Q_10_^Km^ values for depolymerization than for glucose oxidation (Fig. 4a) indicate that the enzymes degrading polymers are more structurally flexible than enzymes oxidizing glucose.

For both polymer decomposition and monomer oxidation, we found stronger thermal adaptation capabilities (i.e. weaker response to temperature differences) for cold- than for warm-adapted soils. We evaluated how thermal adaptation capabilities in microbial communities from different temperature regimes at high (colder climate) and low (warmer climate) altitudes can be explained by three thermal adaptation mechanisms (as described in the introduction).

**Figure 5:**
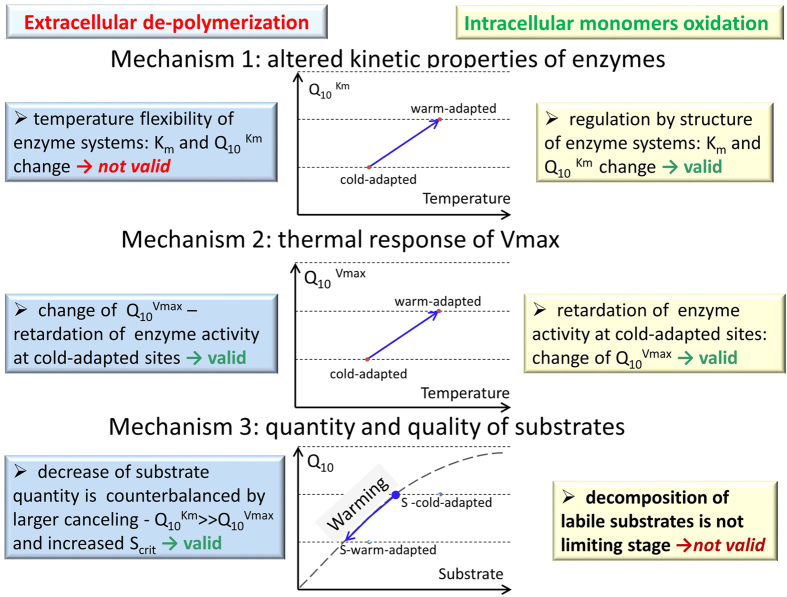
Relevance of three thermal adaptation mechanisms of SOM decomposition based on parameters of enzyme kinetics.

The first mechanism can be identified by different K_m_ values for enzymes produced at different climatic conditions (e.g in elevation gradient). The gradual increase in the affinity (K_m_ decrease) to low-molecular weight substrates at the two highest altitudes (2780 and 3020 m) indicated the occurrence of enzyme isoforms (i.e. of enzymes with similar functions but different substrate affinity and therefore different K_m_^42^,^43^) differing from those at low altitudes (2010 and 2435 m). Such enzyme isoforms can be attributed to a possible shift in microbial community structure^42^,^43^ either due to direct temperature effect or due to temperature induced changes in plant community with corresponding changes in substrate quality. At low altitudes (950, 2010, 2435 m a.s.l.) the K_m_ values were temperature sensitive, demonstrating flexibility of enzyme systems^12^. At high altitudes (2780 and 3020 m), however, the corresponding enzyme isoforms demonstrated no differences in substrate affinity at a 10 °C temperature increase for glucose oxidation (Q_10_ for K_m_ = 1, Fig. 3A, Supplementary Table S4), showing, thus, poor temperature flexibility of intracellular enzymes at high versus low altitudes. The microorganisms at higher altitudes adapted to strong and frequent temperature variations^31^ by increasing substrate affinity and decreasing the temperature flexibility of intracellular enzyme systems (both K_m_ and Q_10_^Km^ decreased). The validity of the first mechanism, therefore, was confirmed for glucose mineralization (Fig. 5).

For polymeric compounds we could not confirm the first mechanism for the two tested sites at elevations differing in 1010 m (Table 1). We found no production of exoenzyme isoforms at increasing altitudes (Fig. 5). Insignificant differences in the K_m_ values at different altitudes indicated that the spectrum of tested exoenzymes was similar (Table 1). Furthermore, the differences in Q_10_^Km^ of hydrolytic enzymes with elevation were insignificant. Our results along the altitudinal gradient on Mt. Kilimanjaro agree with a study^19^ on soils in a continental–scale latitudinal gradient which found no differences in thermal adaptation of K_m_ for 4 of 5 hydrolytic enzymes.

Thus, colder climate with stronger temperature fluctuations did not alter the enzyme systems (K_m_) responsible for decomposition of polymers (Eq. 4). Rather, thermal adaptation at high altitudes was caused by the reduced enzyme pool (E) and/or by lower rates of product release (k_2_) (Eq. 5). This indirectly indicated the validity of the second thermal adaptation mechanism: retarded enzyme production (Fig. 5). Low enzyme concentrations can restrict the absolute values of thermal response of both V_max_ and of overall reaction rate (Q_10_^total^). However, not only absolute but also lower relative response to temperature, i.e. lower Q_10_^total^ and Q_10_^Vmax^ were observed at colder versus warmer climate for both polymer decomposition (Figs 2b and 3B) and for glucose oxidation (Figs 2a and 3B). According to Eq. 5, such a decrease in the Q_10_^Vmax^ means that not only enzyme production decreased, but the rates of product release (k_2_), were also less temperature-sensitive in cold versus warm-adapted soils. Therefore, the increased thermal adaptation for polymer and monomer decomposition with altitude was regulated by a reduced temperature response of V_max_, and decelerated enzyme activity, i.e. by the second mechanism. This is in line with the theoretical model^4^ that explains the mitigation of CO_2_ release from soil in response to warming by a reduced activity of degradative enzymes. Considering that V_max_ governs reaction rates at excess levels of available substrate (Eq. 1) and that labile substrates are very quickly utilized by soil microorganisms^44^, the second mechanism is mainly relevant for short-term responses to warming and for polymer decomposition.

The third proposed mechanism of thermal adaptation refers to the quantity and quality of substrate available for microbial decomposition. In the long-term, a decrease in substrate quantity is stronger under warm than under cold climate^45^. Indeed, a lower amount of labile substrate was found in soils at lower elevations with higher mean temperatures in a similar altitudinal transect at Mt. Kilimanjaro^33^. Lower amount of substrate in warm-adapted soils decreases the reaction rate (Eq. 1), thus restricting temperature response (Fig. 2) and leading to higher thermal adaptation. However, final response of reaction rate to temperature depends both on change in substrate amount and on joint response of V_max_ and K_m_ to temperature increase resulting in canceling effect at substrate concentrations below S_crit_ (Fig. 5). Lower values of S_crit_ indicated weaker canceling (Q_10_^Km^ > Q_10_^Vmax^) and weaker thermal adaptation capabilities of warm versus cold-adapted soils, as was found for polymer decomposition in our study. Thus, trade-off between depletion of available substrate and decrease of S_crit_ values determined the resulting thermal adaptation in warm-adapted soils. Comparing glucose mineralization and extracellular enzymatic activity, we conclude that depolymerization was the main determinant of temperature sensitivity for substrate decomposition. This outlines the importance of high-molecular-weight compounds in thermal adaptation, given that in soil microhabitats the substrate available for decomposition exists mainly as high-molecular-weight polymeric material from plant residues. We emphasized the role of the canceling effect as the main mechanism of thermal adaptation for enzyme-mediated decomposition of polymers. Therefore, global warming will not accelerate the decomposition of plant litter polymers at low concentrations below the relevant S_crit_.

Despite considering relative increases in reaction rate response on temperature (Q_10_), we cannot link the observed differences in kinetic parameters measured in elevation gradient only with climatic (temperature) conditions. Different vegetation types and, consequently, different quality of organic residues also contribute to the temperature sensitivity of enzyme-mediated processes. This calls for the studies separating vegetation and climate (temperature) effects.

Our study revealed the validity of all three thermal adaptation mechanisms. The contributions of these mechanisms to the multistage processes of SOM decomposition vary, depending on the quality and quantity of substrate. Low quality and quantity of substrate not only reduces microbial biomass and potential enzyme activity (V_max_). It also causes a shift in temperature sensitivity of K_m_ and V_max_ for enzyme systems, which can be revealed by the cancelling effect and by associated changes in S_crit_. We therefore suggested S_crit_ as a tool for estimating the substrate concentrations at which decomposition would be insensitive to temperature. The S_crit_ values, however, were larger at higher altitudes, indicating stronger thermal adaptation in cold than in warm-adapted soils, in contrast to common predictions. Thus, thermal adaptation capabilities were mainly governed by stronger temperature fluctuations at higher altitudes and not by the mean annual temperatures. We conclude that the three mechanisms of thermal adaptation are interconnected: cold climate with strong temperature fluctuations caused the changes in the relative temperature sensitivity of the kinetic parameters V_max_ and K_m_, and led to a shift in S_crit_ and a larger canceling effect in cold-adapted soils.

## Methods

### Soil sampling

Soil samples were collected at Mt. Kilimanjaro close to the Machame route (3°4′33″S 37°21′12″E) from five locations. These represented an altitudinal gradient from the colline zone (950 m a.s.l.) to middle subalpine zone (3020 m a.s.l.)^46^ (Table S1). At each location, 5 soil cores (2.5 cm diameter × 5 cm depth) were taken individually, transported to the laboratory unfrozen and mixed similarly to^19^. Because the soil moisture was very low, the soils were pre-moistened up to 40% of WHC and pre-conditioned at corresponding temperatures (10 and 20 °C) for two days before enzyme activity measurements.

### Enzyme activity

Activities of extracellular hydrolytic enzymes were determined at 10 and 20 °C using fluorogenically labelled substrates^47^,^48^. We used 10 and 20 °C temperatures to standardize the comparison of different sites. It was considered that 10 °C is a common temperature for all sites tested^32^, while 20 °C is common for the sites up to 2500 m. Even for the sites at highest altitudes (2780 m and 3020 m) a temperature of 20 °C is probable during summer. We therefore assumed that, at all altitudes, microorganisms were accustomed to both temperatures. We further assumed that adaptive mechanisms were different at low vs high altitudes, causing differences in temperature sensitivity of enzyme systems. The potential enzymatic activity was estimated for all 5 altitudes studied, using saturating concentrations of substrate that were determined in preliminary tests. Thereafter, two sites (2010 and 3020 m) with moderate difference in C content and 10 °C difference in MAT were chosen for detailed investigation of enzyme kinetics. As compared with the site at 2010 m, the site at 3020 m was characterized by up to 1.7 and 2.2 times stronger seasonal and diurnal temperature fluctuations, respectively^32^. The sites with highest (2780 m, C_org_ 20.9%) and the lowest (2435 m, C_org_ 10.7%) C_org_ content as well as the site with highest MAT and very different vegetation (950 m, MAT 22 °C, tropical forest) were not included in this detailed assessment.

Three fluorogenic enzyme substrates based on 4-methylumbelliferone (MUF) were used to assess activity: MUF-β-D-glucopyranoside (EC 3.2.1.21) for β-glucosidase, MUF-N-acetyl-β-D-glucosaminide dehydrate (3.2.1.14) for N-acetylglucosaminidase, and 4-MUF-phosphate (EC 3.1.3.2) for acid phosphatase. The calibration solutions were prepared using soil suspension and a gradient of MUF concentrations (0–100 μM). Calibration curves as well as the controls for the autofluorescence of the substrate and for the quenching effect were included in every series of enzyme measurements. Fluorescent substrates were added to the assay wells in concentrations of 1–300 μM for phosphatase and 1–150 μM for other enzymes. The highest concentrations of 4-MUF-phosphate and of other substrates corresponded, respectively, to 55 and 28 μmol g^−1^ soil. Fluorescence was measured each 30 min during 2 h incubations of soil suspension with fluorogenic substrates at an excitation wavelength of 355 nm and an emission wavelength of 460 nm, split width of 25 nm, with a Victor 1420–050 Multilabel Counter (Perkin Elmer, Waltham, USA). Enzyme activity was calculated from the initial linear increase in MUF with time and was expressed in μmol per g soil per hour (μmol g^−1^ h^−1^).

### ^14^C glucose

Subsamples of soil (1 g) were placed in 24-well microplates, which were specially designed for ^14^CO_2_ trapping from small amounts of soil. ^14^CO_2_ was trapped by 1 N NaOH placed in a neighbour well, which was connected to the well containing soil. Then 100 μl of ^14^C(U)D-glucose solution was added at a rate of 2–200 μg C g^−1^, corresponding to an activity of 5.8 × 10^3^ Bq. The appropriate concentration interval was determined for each treatment in preliminary experiments (data not shown). Immediately after adding ^14^C-glucose, the microplate was tightly sealed with a plastic cover. The microplates were then incubated at 10 and 20 °C for 20 min, then the reaction was stopped by adding 6 M H_3_PO_4_, which ensured complete ^14^CO_2_ evolution from soil pores.

The ^14^C activity collected as ^14^CO_2_ with NaOH was measured in 1 ml of the scintillation cocktail Rotiszint Eco Plus (Carl Roth, Karlsruhe, Germany) after decay of the chemiluminescence. ^14^C activity was measured using a Wallac 1411 Liquid Scintillation Counter (Wallac Oy, Turku, Finland). The ^14^C counting efficiency was 89% and the ^14^C activity measurement error did not exceed 2%. The absolute ^14^C activity was standardized by adding NaOH solution as a quencher to the scintillation cocktail and using the spectrum of an external standard (SQP(E) method).

### Statistical analysis and modelling

The means of three replicates with standard deviations are presented in tables and figures. The parameters of Equation 1 were fitted by minimizing the least-square sum using ModelMaker software Version 3.0.3^49^. The significant effects of temperature on parameters of enzyme kinetics were assessed by one-way ANOVA at P < 0.05.

## Additional Information

**How to cite this article**: Blagodatskaya, E. *et al.* Temperature sensitivity and enzymatic mechanisms of soil organic matter decomposition along an altitudinal gradient on Mount Kilimanjaro. *Sci. Rep.* **6**, 22240; doi: 10.1038/srep22240 (2016).

## Supplementary Material

Supplementary Information

## Figures and Tables

**Table 1:** Temperature-induced changes in enzyme affinity to substrate (K_m_) and in maximal reaction rate (V_max_) and activation energy (E_a_) for hydrolytic enzymes in soils from 2010 and 3020 m a.s.l. on Mt. Kilimanjaro.

Altitude	Temperature	Glucosidase			Chitinase			Phosphatase		
		K_m_*	V_max_	E_a_	K_m_	V_max_	E_a_	K_m_	V_max_	E_a_
2010 m	10 °C	2.27^b^ ± 0.28	1.43^c^ ± 0.05	57.0	3.88^b^ ± 0.28	0.67^c^ ± 0.02	57.5	8.24^b^ ± 0.90	1.14^d^ ± 0.05	84.6
	20 °C	7.46^a^ ± 0.54	3.29^a^ ± 0.09		11.85^a^ ± 1.64	1.57^a^ ± 0.1		28.32^a^ ± 2.13	3.77^b^ ± 0.13	
3020 m	10 °C	2.93^b^ ± 0.38	1.64^b^ ± 0.05	46.2	4.14^b^ ± 0.57	0.57^d^ ± 0.03	35.0	9.13^b^ ± 1.24	3.03^c^ ± 0.15	39.1
	20 °C	8.1^a^ ± 0.37	3.23^a^ ± 0.06		8.89^a^ ± 1.36	‘0.96^b^ ± 0.09		25.99^a^ ± 1.8	6.46^a^ ± 0.25	

Within each column, values marked by the same letters are not significantly different.

^*^K_m_ values in μmol MUF g^−1^ soil, and V_max_ values in μmol MUF g^−1^ soil h^−1^, E_a_ – in kJ mol^−1^.
