# Supplementary material for: Temperature sensitivity and enzymatic mechanisms of soil organic matter decomposition along an altitudinal gradient on Mount Kilimanjaro
Source: Sci Rep. 2016 Feb 29;6:22240. doi: 10.1038/srep22240 (PMC4770592; doi:10.1038/srep22240)
Supplement: Supplementary Information [file srep22240-s1.pdf]

Supplementary material

Type of contribution: Article

Temperature sensitivity and enzymatic mechanisms of soil organic matter decomposition along an altitudinal gradient on Mount Kilimanjaro

Authors:

Evgenia Blagodatskaya[1,2,6*], Sergey Blagodatsky[2,3], Nikita Khomyakov[4], Olga Myachina[5], Yakov Kuzyakov[1,6]

[1] Dept. of Soil Science of Temperate Ecosystems, University of Göttingen, 37077 Göttingen, Germany

[2] Institute of Physicochemical and Biological Problems in Soil Science, Russian Academy of Sciences, 142290 Pushchino, Russia

[3] Institute of Plant Production and Agroecology in the Tropics and Subtropics, University of Hohenheim, 70593 Stuttgart, Germany

[4] Forensic Science Centre of the Ministry of Internal Affairs of the Russian Federation, 125130 Moscow Russia

[5] Institute of General and Inorganic Chemistry, 100170 Tashkent, Uzbekistan

[6] Department of Agricultural Soil Science, University of Göttingen, 37077 Göttingen, Germany

Corresponding author: Evgenia Blagodatskaya

phone: +495513920509

Email: janeblag@mail.ru

Correspondence address:

Dept. of Soil Science of Temperate Ecosystems, University of Göttingen, 37077 Göttingen, Germany

Supplementary table S1. Characteristics of sampling sites and soils from altitudinal transect on Mt. Kilimanjaro, MAT – mean annual temperature.

Altitude, m a.s.l.	Altitudinal zone	Vegetation type	C_{org}	N	C/N	pH	MAT °C
			%	%			
950	colline zone	low mountain tropical forest	12.8	0.9	14.2	4.5	22
2010	middle mountane zone	*Ocotea-Podocarpus* forest	15.2	0.87	17.5	4.0	17.5
2435	upper mountane zone	*Podocarpus* forest	10.7	0.62	17.3	4.5	12.5
2780	lower subalpine zone	*Erica excelsa* / *Podocarpus* forest	20.9	1.2	17.4	3.7	8.5
3020	middle subalpine zone	*Erica excelsa* / Erica shrubland	11.6	0.7	16.6	4.3	7.2

Supplement 2. Derivation of $Q_{10total}$ and S_{crit}.

Both V_{max} and K_m are responsible for the reaction rate (v) in Michaelis–Menten kinetics. As V_{max} increases with increasing temperature, the reaction rate can potentially increase, but as K_m also increases with increasing temperature, the affinity for the substrate decreases, so the reaction is slowed.

If a canceling effect occurs, the temperature increase does not cause the reaction rate to increase. Let the 10 °C shift in temperature cause $\mathbf{Q_{10}^{Vmax}}$-fold increase in V_{max} and $\mathbf{Q_{10}^{Km}}$-fold increase in K_m. Then the reaction rates at 10 °C: $\mathbf{v}^{10oC} = V_{max}*S/(K_m+S)$

and at 20 °C: $\mathbf{v}^{20oC} = \mathbf{Q_{10}^{Vmax}} *V_{max}*S/(\mathbf{Q_{10}^{Km}} *K_m+S)$

Accordingly, the Q_{10} of reaction rate ($\mathbf{Q_{10}^{total}}$) is derived as:

$\mathbf{Q_{10}^{total}} = \mathbf{v}^{20oC}/\mathbf{v}^{10oC} = \mathbf{Q_{10}^{Vmax}} *V_{max}*S*(K_m+S)/(\mathbf{Q_{10}^{Km}} *K_m+S)*V_{max}*S$,

Finally, $\mathbf{Q_{10}^{total}} = \mathbf{(Q_{10}^{Vmax}} *(K_m+S))/(\mathbf{Q_{10}^{Km}} *K_m+S)$ (1S)

Canceling means: $\mathbf{v}^{10oC} = \mathbf{v}^{20oC}$, i.e.

$V_{max}*S/(K_m+S) = \mathbf{Q_{10}^{Vmax}} *V_{max}*S/(\mathbf{Q_{10}^{Km}} *K_m+S)$ (2S)

We transformed Eq.1S to analyze how high the $\mathbf{Q_{10}^{Km}}$ (i.e. the K_m increase) should be to counterbalance a V_{max} increase by the factor of $\mathbf{Q_{10}^{Vmax}}$:

$\mathbf{Q_{10}^{Km}} = (\mathbf{Q_{10}^{Vmax}}\ K_m\ )/\ K_m + (\mathbf{Q_{10}^{Vmax}}\ -1)*S)/K_m$, or

$$\mathbf{Q_{10}^{Km}} = \mathbf{Q_{10}^{Vmax}} + (\mathbf{Q_{10}^{Vmax}} -1)*S/K_m. \qquad (3S)$$

The substrate concentration in Equation 2S equals the critical substrate concentration (S_{crit}) below which the canceling effect occurs. Theoretically, three situations can occur as a response of Michaelis-Menten parameters to temperature increase:

1) both K_m and V_{max} increase equally: $S_{crit} = (\mathbf{Q_{10}^{Km}} - \mathbf{Q_{10}^{Vmax}})*K_m/\ (\mathbf{Q_{10}^{Vmax}} -1)$. If $\mathbf{Q_{10}^{Km}} = \mathbf{Q_{10}^{Vmax}}$, canceling effect can occur only at zero substrate concentrations;
2) K_m increase is weaker than V_{max}: $\mathbf{Q_{10}^{Km}} < \mathbf{Q_{10}^{Vmax}}$. Canceling effect can occur only if $\mathbf{Q_{10}^{Vmax}} < 1$, i.e. both V_{max} and K_m decrease with temperature increase;
3) K_m increase is greater than V_{max}: $\mathbf{Q_{10}^{Km}} > \mathbf{Q_{10}^{Vmax}}$. Canceling effect can occur at $S_{crit} = [(\mathbf{Q_{10}^{Km}} - \mathbf{Q_{10}^{Vmax}})/\ (\mathbf{Q_{10}^{Vmax}} - 1)] * K_m$

Theoretically it is possible **that a temperature increase even results in slower reaction rates** for enzyme kinetics at low substrate concentrations:

$\mathbf{v}^{20oC} / \mathbf{v}^{10oC} = \mathbf{Q_{10}^{Vmax}}\ (K_m+S) / (\mathbf{Q_{10}^{Km}}\ K + S) < 1$,

$S < [(\mathbf{Q_{10}^{Km}} - \mathbf{Q_{10}^{Vmax}}) / (\mathbf{Q_{10}^{Vmax}} -1)] * K_m$

This relationship is valid only for greater temperature sensitivity of K_m versus V_{max}. ($\mathbf{Q_{10}^{Km}} > \mathbf{Q_{10}^{Vmax}}$).

At $S > [(\mathbf{Q_{10}^{Km}} - \mathbf{Q_{10}^{Vmax}}) / (\mathbf{Q_{10}^{Vmax}} -1)] * K_m$ the positive temperature response, i.e. increase in mineralization rates with the temperature can be predicted based on Michaelis–Menten kinetics.

Supplementary table S3. Substrate amounts below which a canceling effect occurs calculated by Eq.2 for the activity of hydrolytic enzymes in soils from 2010 and 3020 m a.s.l. on Mt. Kilimanjaro

Altitude	S_{crit} Substrate, µmol g^{-1}		
	Glucosidase	Chitinase	Phosphatase
2010 m	1.68	2.15	0.46
3020 m	2.39	2.90	5.71

Supplementary table S4. Temperature-induced changes in substrate-to-enzyme affinity (K_m), in maximal rate (V_{max}) and activation energy (E_a) of glucose oxidation in soils from an altitudinal transect on Mt. Kilimanjaro. The data marked by same letters (case-specific) are not significantly different.

Altitude, ma.s.l.	K_m, µg C g^{-1}		V_{max}, µg CO_2-C g^{-1} h^{-1}		E_a, kJ mol^{-1}
	10°C	20°C	10°C	20°C	
950	$6.38^d \pm 0.53$	$20.80^c \pm 1.36$	$2.13^J \pm 0.01$	$9.30^D \pm 0.21$	100.4
2010	$17.67^c \pm 2.18$	$29.39^b \pm 1.48$	$2.37^H \pm 0.09$	$12.24^C \pm 0.21$	111.9
2435	$17.49^c \pm 3.1$	$34.05^a \pm 2.84$	$2.18^{HJ} \pm 0.11$	$5.49^F \pm 0.13$	62.9
2780	$7.34^d \pm 1.64$	$7.31^d \pm 0.49$	$14.84^B \pm 1.39$	$32.29^A \pm 2.24$	53.0
3020	$3.12^e \pm 0.43$	$3.13^e \pm 0.25$	$3.02^G \pm 0.07$	$7.98^E \pm 0.12$	66.1

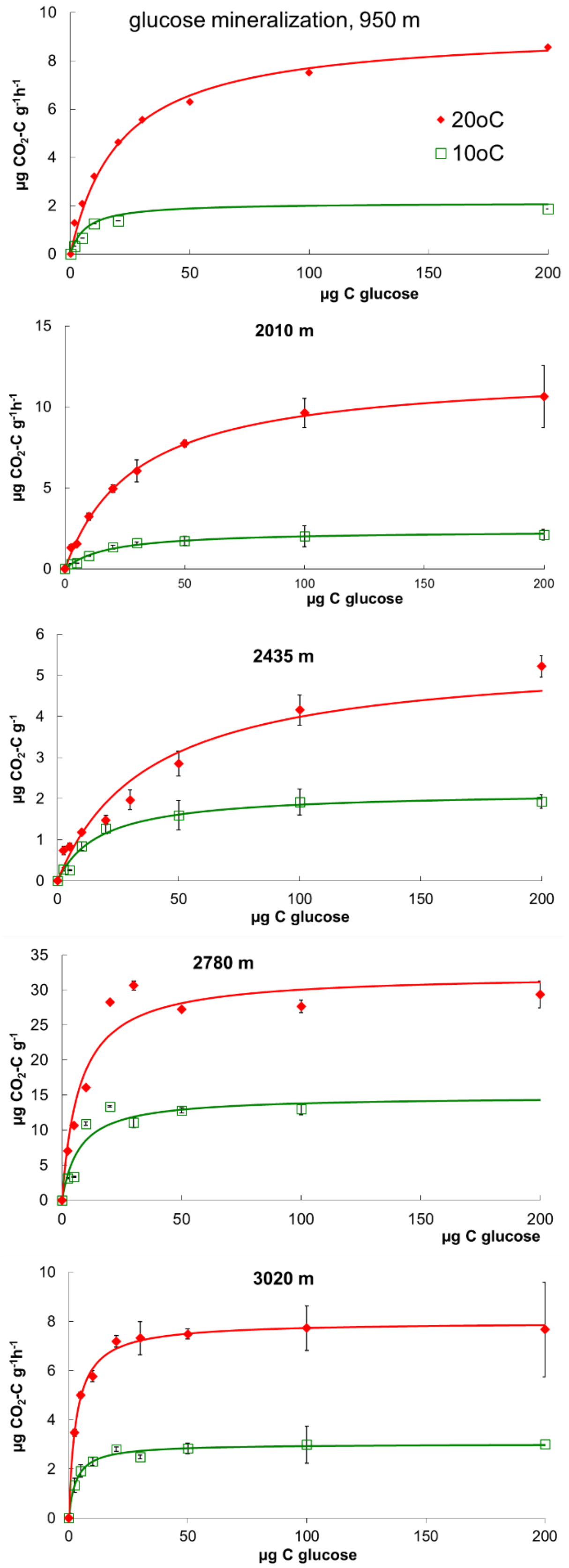

Supplementary Figure S5. Rate of CO_2-C production during glucose oxidation as dependent on substrate concentration at 10 and 20 °C for different altitudes. Symbols – experimental data, lines – approximation by Michaelis–Menten kinetics. Bars show standard deviations of the means (n=3).
